# Effectiveness of Periodic Repolishing on the Surface and Mechanical Properties of Resin‐Based CAD/CAM Materials After Different Cycles of Combined Simulated Toothbrushing and Mechanical Cycling

**DOI:** 10.1111/jerd.70235

**Published:** 2026-07-19

**Authors:** Gabriel Nunes Sampaio, Amanda Martinelli Moretti, Mariana Nogueira Bianchi, Victor Alves Nascimento, Aldiéris Alves Pesqueira

**Affiliations:** ^1^ Department of Dental Materials and Prosthodontics São Paulo State University (UNESP), School of Dentistry Araçatuba Brazil

**Keywords:** CAD/CAM blocks, fatigue, flexural strength, hardness, repolishing, surface roughness, toothbrushing simulation

## Abstract

**Objective:**

This study aimed to evaluate the effectiveness of periodic repolishing on the surface and mechanical properties of resin‐based CAD/CAM materials subjected to combined mechanical cycling and simulated toothbrushing, with or without periodic repolishing.

**Methods:**

Two resin‐based CAD/CAM materials were investigated (Tetric CAD [TC] and Shofu HC [SH]). Groups (*n* = 12) were assigned according to the evaluation period and repolishing (RPL) condition. The aging protocol comprised simulated toothbrushing (ST—200 g load, 250 cycles/min) combined with mechanical cycling (MC—50 N load, 1.67 Hz frequency) performed in aging intervals (AI—2.4 × 10^5^ MC + 10,000 ST), totaling two periods (5 × AI and 10 × AI). Periodic repolishing was performed using a clinical polishing system (OptraGloss, Diamond High‐Gloss polisher [HP]). Surface roughness (Ra), Vickers microhardness (VH), flexural strength (FS), and elastic modulus (EM) were quantitatively evaluated, while SEM and AFM analyses were performed qualitatively. Data normality and homogeneity were verified, followed by two‐way ANOVA and Tukey's post hoc test (*α* = 0.05).

**Results:**

Aging significantly increased surface roughness and reduced microhardness in both resin‐based CAD/CAM materials (*p* < 0.05). Periodically repolished groups (T_1RPL_ and T_2RPL_) showed higher VH and lower Ra values (*p* < 0.05) than their non‐repolished counterparts (T_1_ and T_2_). No significant differences in FS or EM were observed after aging (*p* = 0.664 and 0.185, respectively).

**Conclusion:**

Aging significantly affected the surface roughness and microhardness of both materials. Periodic repolishing proved to be an effective strategy for minimizing the effects of aging, thereby reducing surface roughness and preserving microhardness.

**Clinical Significance:**

Intraoral repolishing at yearly recall intervals may help control surface degradation, maintain smoother surfaces, and preserve microhardness over time, particularly for indirect restorations fabricated from resin‐based CAD/CAM materials.

## 1 Introduction

Oral rehabilitation has been widely performed using the subtractive method through CAD/CAM systems, which enable the milling of different restorative materials with high precision, predictability, and clinical efficiency [1, 2, 3, 4]. In this context, in addition to ceramic materials, resin‐based blocks specifically developed for this milling method have emerged as an alternative restorative option [2, 3, 4].

The presence of a resin matrix associated with ceramic fillers in these materials has resulted in important advantages. These materials are easier to mill, reducing tool wear during manufacturing and decreasing the occurrence of marginal chipping during the fabrication of restorations [5]. In addition, they exhibit lower brittleness and an elastic modulus closer to that of dental structures, thereby reducing the risk of fractures [5, 6, 7, 8, 9, 10]. Another relevant aspect is the optimization of the clinical workflow, since these materials do not require additional steps, such as sintering, crystallization, or firing [11]. This characteristic favors their use in chairside protocol, in which the restoration is fabricated and installed in a single session [12]. On the other hand, compared to ceramics, these materials have lower flexural strength, elastic modulus, fracture toughness, and surface hardness [13, 14, 15, 16]. Although lower stiffness may contribute to greater elastic deformation capacity and stress absorption, these characteristics may also represent limitations in situations of high occlusal stress or in extensive restorations. Furthermore, the presence of a resin matrix makes these materials potentially more susceptible to physicochemical degradation, water absorption, and intrinsic aging, with possible impairment of surface, mechanical, and optical properties over time [17, 18, 19, 20].

In view of this, it is important that restorations present smooth and well‐polished surfaces, as this is closely related to their clinical longevity [21, 22, 23, 24, 25], especially for materials with lower abrasion resistance [12, 13, 14, 15, 16, 17]. To achieve this goal, several mechanical polishing protocols, both laboratory and clinical (chairside polishing), have been proposed for resin‐based CAD/CAM materials, including the use of abrasive rubber polishers and discs [26, 27]. Thus, single‐step polishing systems have gained prominence due to their practicality and time efficiency, becoming increasingly popular in clinical practice [28]. This mechanical polishing approach is effective not only for extraoral polishing, but also allows intraoral polishing after occlusal and interproximal adjustments, as well as repolishing during periodic maintenance visits. This represents a clinically relevant but systematically underexplored strategy, given that this practice may contribute to the recovery of microhardness and to the reduction of surface roughness values considered clinically unacceptable after aging [29, 30], playing a fundamental role in preserving the properties of restorative materials throughout clinical use.

Furthermore, the simple process of daily toothbrushing may cause morphological changes and an increase in the surface roughness of ceramics and composite materials [31, 32, 33], especially when associated with the use of abrasive toothpastes, thereby accelerating wear, surface porosity, color alteration, and water absorption [32, 34, 35, 36]. In this context, simulated mechanical brushing has been widely employed as an appropriate method to evaluate the impact of oral hygiene habits on the surface characteristics of restorative materials [37]. In addition, restorations are continuously exposed to cyclic masticatory forces in a moist environment [38], which can result in a significant reduction in their mechanical properties.

Although previous studies [12, 39, 40], have compared different polishing kits for the mechanical polishing of restorations made from resin‐based CAD/CAM materials, there remains no consensus on the most effective technique or instrument for reducing the roughness of these materials. The literature highlights the need for further research to better understand the advantages and limitations of resin‐based CAD/CAM materials, as well as to define more effective protocols for polishing and maintaining their properties. In addition, inconsistent results regarding clinical finishing and polishing systems, due to variables such as the materials tested and the evaluation methods employed, hinder the establishment of a universally effective protocol [40, 41, 42].

Therefore, it is essential to investigate the effects of aging and the effectiveness of polishing protocols, with particular attention to periodic repolishing, on the surface and mechanical properties of resin‐based CAD/CAM materials. Elucidating these aspects may represent a clinically relevant but systematically underexplored approach to increase the durability of restorations, as well as providing scientific evidence to support the development of more effective and evidence‐based clinical protocols. Thus, the aim of this study was to evaluate the effectiveness of periodic repolishing on the surface and mechanical properties of resin‐based CAD/CAM materials subjected to mechanical polishing as a surface treatment and subsequently exposed to different cycles of mechanical cycling (MC) associated with simulated toothbrushing (ST), with or without periodic repolishing procedures. The research hypothesis was that periodic repolishing would influence the surface and mechanical characteristics of aged resin‐based CAD/CAM materials tested.

## 2 Materials and Methods

### 2.1 Sample Size Calculation

An a priori sample size calculation was performed using G*Power software (v3.0.10, Franz Faul, Universität Kiel, Germany), based on effect size estimates from a pilot study, with a significance level of *α* = 0.05 and a statistical power of 80% (1 − *β*). The minimum sample size was *n* = 12 for the flexural strength test, with smaller sample sizes estimated for the other analyses. However, to standardize the experimental design and increase statistical robustness, the largest calculated sample size (*n* = 12) was adopted for all analyses.

### 2.2 Specimen Preparation

For each of the two resin‐based CAD/CAM materials [Tetric CAD (TC), Ivoclar Vivadent, Schaan, Liechtenstein; Shofu HC (SH), Shofu Inc., Kyoto, Japan], bar‐shaped specimens (*n* = 60) measuring 4.0 × 2.0 × 17.0 mm [43] were fabricated using a precision metallographic cutting machine (Isomet 1000, Buehler, Illinois, USA). The materials are listed and described in Table 1. After cutting, each specimen was polished with the light blue, single‐step Diamond High‐Gloss polisher (HP) from the OptraGloss kit (Ivoclar Vivadent AG, Schaan, Liechtenstein), using a low‐speed contra‐angle handpiece, applying moderate pressure with constant linear movements at 10,000 rpm for 3 min. A single operator performed this procedure to ensure standardization of the process [28]. Surface roughness was measured to verify that standardization among specimens of each material had been achieved. In addition, graphical analysis (Figure 1) and equivalence testing (Table 2) were performed to confirm the comparability of the initial roughness values among the experimental groups. After polishing, the specimens were cleaned in an ultrasonic bath with water and then air‐dried.

**TABLE 1:** Description of the materials used in the study.

Code—product name	Composition			Shade—batch number	Manufacturer
	Monomer	Filler weight %	Filler		
TC—Tetric CAD	Bis‐GMA, UDMA, Bis‐EMA, TEGDMA	71	Barium glass (< 1 μm) and silicon dioxide (< 20 nm)	A2—Z045WB	Ivoclar Vivadent AG
SH—Shofu HC	UDMA, TEGDMA	61	Silica powder, microfumed silica, and zirconium silicate	A2—0819959	Shofu Inc.
HP—Diamond High‐Gloss polisher	Diamond material, dicyclohexylmethane‐4,4′‐diisocyanate, diethylmethylbenzenediamine, titanium dioxide			ZL09JP	Ivoclar Vivadent AG

Abbreviations: Bis‐EMA = ethoxylated bisphenol A dimethacrylate; Bis‐GMA = bisphenol A glycol dimethacrylate; Bis‐MEPP = bisphenole A ethoxylate dimethacrylate; TEGDMA = Triethylene glycol dimethacrylate; UDMA = Urethane dimethacrylate.

**FIGURE 1:**
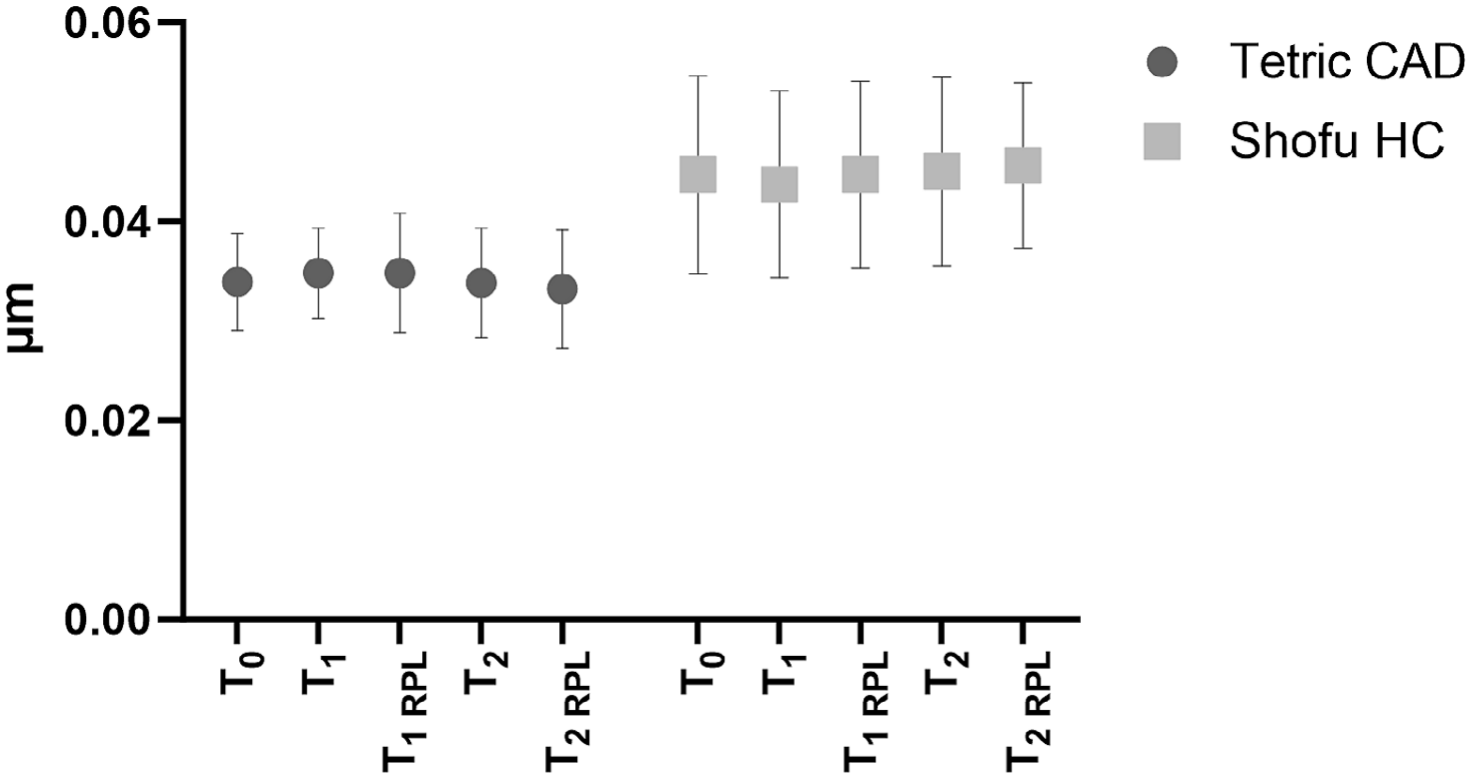
Mean values and standard deviations for initial surface roughness (Ra) for the experimental groups after the standardized polishing procedure.

**TABLE 2:** One‐way ANOVA results for the comparison of surface roughness (Ra) among the experimental groups after the standardized polishing procedure.

	*F*	df1	df2	*p*
Tetric CAD	0.545	4	55	0.704
Shofu HC	0.0608	4	55	0.993

### 2.3 Experimental Groups and Aging Protocol

Aging consisted of combined mechanical cycling (MC) and simulated toothbrushing (ST). The aging cycles were defined as aging intervals (AI = MC + ST), each comprising 2.4 × 10^5^ mechanical cycles and 10,000 simulated toothbrushing cycles. This protocol was adopted so that each AI corresponded to 1 year of clinical service [43, 44, 45].

After polishing, the specimens were randomly allocated (Excel; Microsoft Corp., Redmond, WA, USA) to groups corresponding to the evaluation periods. Five experimental groups were established for each material (T_0_; T_1_; T_1RPL_; T_2_; T_2RPL_). The T_0_ group corresponded to the initial condition without aging. These specimens were stored in distilled water for 24 h before baseline evaluations. For the first aging period (T_1_), specimens were subjected to 5 × AI, whereas for the second aging period (T_2_), specimens underwent 10 × AI, simulating 5 and 10 years of clinical use, respectively. The groups subjected to periodic repolishing during aging, corresponding to their non‐repolished counterparts (T_1_ and T_2_), were designated as repolishing (RPL) groups (T_1RPL_ and T_2RPL_). In these groups, specimens underwent a repolishing procedure identical to the initial polishing protocol at the end of each aging interval (AI). The detailed correspondence between the experimental groups and the aging protocols is presented in Figure 2.

**FIGURE 2:**
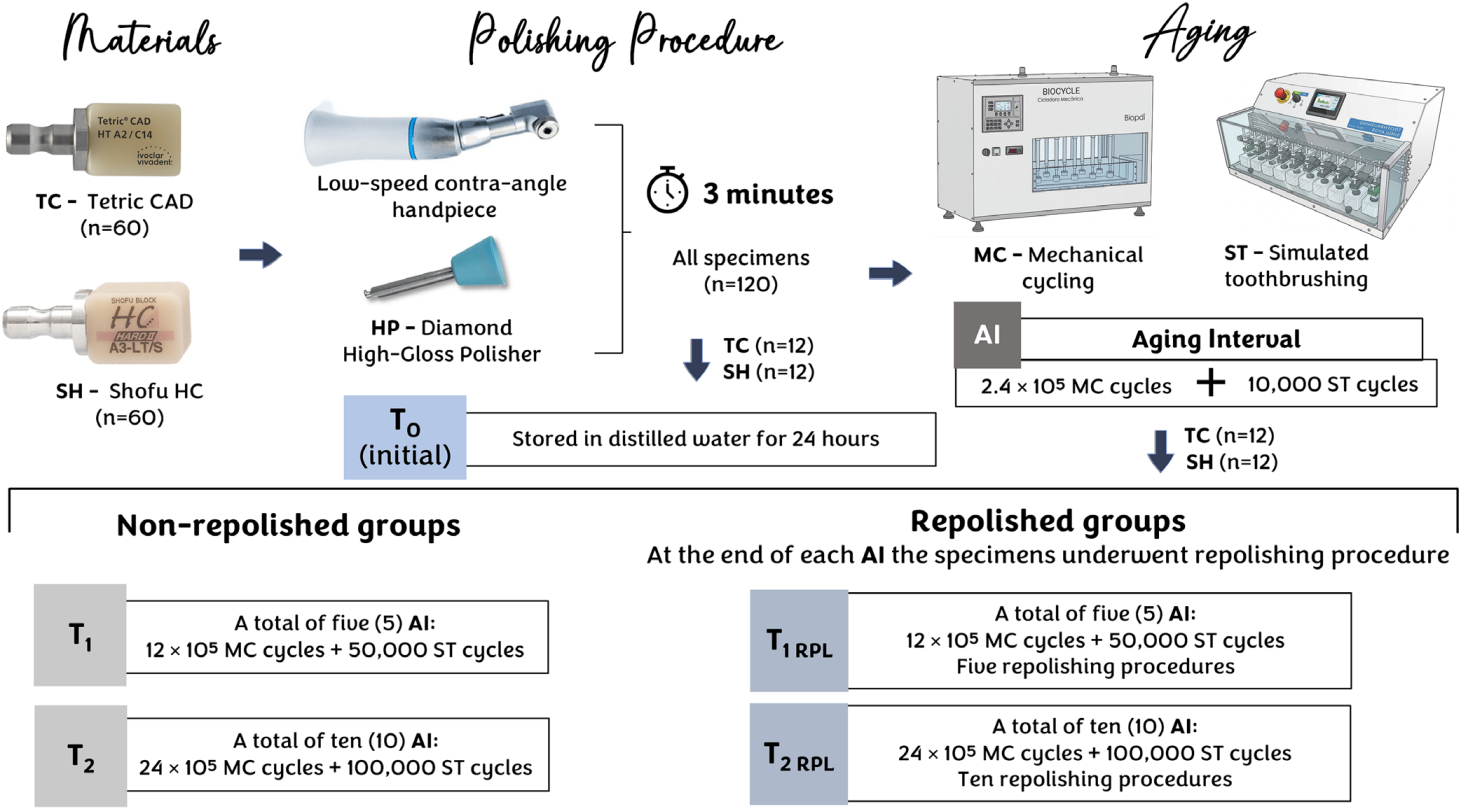
Flowchart containing detailed description of the correspondence between the experimental groups and the aging protocols.

An additional double‐polished control group (DPC) was included for surface roughness and Vickers microhardness analyses to evaluate the possible cumulative effect of repeated polishing on surface‐related outcomes. In this group, specimens received the same initial polishing protocol followed by successive repolishing procedures without intervening aging. Two DPC groups were established according to the number of repolishing procedures performed in the aged repolished groups: T_1DPC_ and T_2DPC_.

For the MC procedure, the specimens were immersed in distilled water and subjected to a 50 N load at a frequency of 1.67 Hz [43] using an accelerated fatigue machine (Biocycle V2, BIOPDI, São Carlos, SP, Brazil). A corrosion‐resistant steel indenter with a diameter of 8 mm served as the antagonist, applying a perpendicular force at the center of the bar‐shaped specimen, which was supported on a silicone matrix within metallic bases.

After MC, the specimens were subjected to ST on the same previously cycled surface to simulate daily abrasive wear associated with oral hygiene. The simulated toothbrushing test was conducted using a brushing machine (Elquip Maq Escovação, São Carlos, Brazil) equipped with a motor that moved the arms in a back‐and‐forth motion under a 200 g load at 250 cycles per minute [44, 45]. The brushing slurry consisted of a mixture of Colgate Total 12 dentifrice (Colgate‐Palmolive, São Paulo, SP, Brazil) and distilled water in a 1:2 weight ratio [44].

### 2.4 Surface Roughness

Surface roughness (Ra) was measured using a portable profilometer (SJ‐401, Mitutoyo Sul Americana Ltda., Santo Amaro, SP, Brazil). Three measurements were obtained at the center and both ends of each specimen, and the mean value was calculated. The parameters used were a cut‐off of 0.25 mm and a measuring speed of 0.5 mm/s [46].

### 2.5 Mechanical Properties

The surface microhardness of the specimens was evaluated using a Vickers Microhardness tester (HMV‐2000, Shimadzu Corp., Tokyo, Japan) under a 0.98‐N load for 15 s. Three indentations were performed at the center and both ends of each specimen, and the mean value was calculated. The Vickers hardness (VH) values were calculated using the standard equation VH = 0.18544 × (*P*/*d* ^2^), where *P* is the applied load (N) and *d* is the mean length of the indentation diagonals (mm) [47].

After the other tests, the specimens were subjected to a three‐point bending test to determine flexural strength (FS) using a universal testing machine (DL 2000, EMIC, São José dos Pinhais, PR, Brazil). The support span was set at 12 mm, and the crosshead speed was 1 mm/min. Specimens were positioned with the previously aged surface facing upward. Flexural strength (MPa) was calculated using the following equation: *σ* = 3*Pl*/2*bd* ^2^, where *P* = maximum load (N), *l* = span length (mm), *b* = specimen width (mm), and *d* = specimen thickness (mm). The exact dimensions of each specimen were measured at the midpoint using a digital caliper (Digimatic Digital Caliper, Mitutoyo Sul Americana Ltda, Santo Amaro, SP, Brasil) [47].

To obtain the elastic modulus (EM) values, the final flexural strength test data were used in the following equation: *E* = *Fl* ^3^/4*bh* ^3^ *d*. Where *F* is the load (N) at a point in the straight‐line portion (with the maximum slope) of the load/deflection curve, *d* is the deflection (mm) at load *F*, *h* is the specimen thickness (mm), and *b* and *l* are as defined above [47].

### 2.6 Surface Characterization

One additional specimen from each experimental group of each material (*n* = 1) was used for SEM and AFM analyses. Prior to SEM evaluation, the specimens were sputter‐coated for 30 s using a metallization device (Quorum Q150R ES, Quorum Technologies, East Sussex, UK). Scanning electron micrographs (EVO LS15, Carl Zeiss, Oberkochen, Germany) were obtained at 1000× and 10,000× magnifications, with accelerating voltages of 20 or 30 kV, depending on the imaging conditions. Surface topography was evaluated using an atomic force microscope (NanoObserver III, Concept Scientific Instruments, Les Ulis, France) equipped with a Tap190Al‐G tip. Two‐dimensional (2D) and three‐dimensional (3D) images (20 × 20 μm) were obtained during AFM analysis using the true non‐contact mode at a scan rate of 0.75 Hz. Gwyddion software (v2.69; Czech Metrology Institute, Brno, Czech Republic) was used to process the images.

### 2.7 Data Analysis

Statistical analyses were performed using statistical software (Jamovi, version 2.6.26, The jamovi project, Sydney, Australia). Data normality and homogeneity of variances were evaluated using the Shapiro–Wilk and Levene tests, respectively. A two‐way analysis of variance (ANOVA) was performed to assess the effects of the factors and their interaction. When necessary, the Tukey post hoc test was used for multiple comparisons. The significance level was set at *α* = 0.05.

## 3 Results

The descriptive values for all evaluated outcomes, according to material and experimental group, are presented as mean ± standard deviation in Table S1.

For both surface roughness (Table 3) and Vickers microhardness (Table 4), two‐way analysis of variance (ANOVA) revealed significant effects of material and evaluation period (*p* < 0.001), as well as a significant interaction between these factors (*p* < 0.001).

**TABLE 3:** Two‐way ANOVA results for surface roughness.

	Sum of squares	df	Mean square	*F*	*p*	*η* ^2^
Material	0.00523	1	0.00523	55.5	< 0.001	0.018
Evaluation period	0.24792	6	0.04132	438.5	< 0.001	0.875
Material × evaluation period	0.01417	6	0.00236	25.1	< 0.001	0.050

**TABLE 4:** Two‐way ANOVA results for Vickers microhardness.

	Sum of squares	df	Mean square	*F*	*p*	*η* ^2^
Material	12,569	1	12568.87	5101.20	< 0.001	0.879
Evaluation period	1274	6	212.30	86.17	< 0.001	0.089
Material × evaluation period	137	6	22.86	9.28	< 0.001	0.010

The multiple‐comparison test showed that SH exhibited higher Ra values than TC only at T_0_ (*p* < 0.05), whereas lower Ra values were observed at all other evaluation periods (*p* < 0.05). For both materials, Ra was significantly higher at T_1_ and T_2_ than at T_0_, with the highest value observed in the last evaluation period (*p* < 0.05). In contrast, periodic repolished groups (T_1RPL_ and T_2RPL_) did not differ from T_0_ for TC (*p* = 0.635 and *p* = 1.000), whereas even lower Ra values were observed for SH (*p* < 0.05). The data and comparisons for surface roughness are presented in Figure 3.

**FIGURE 3:**
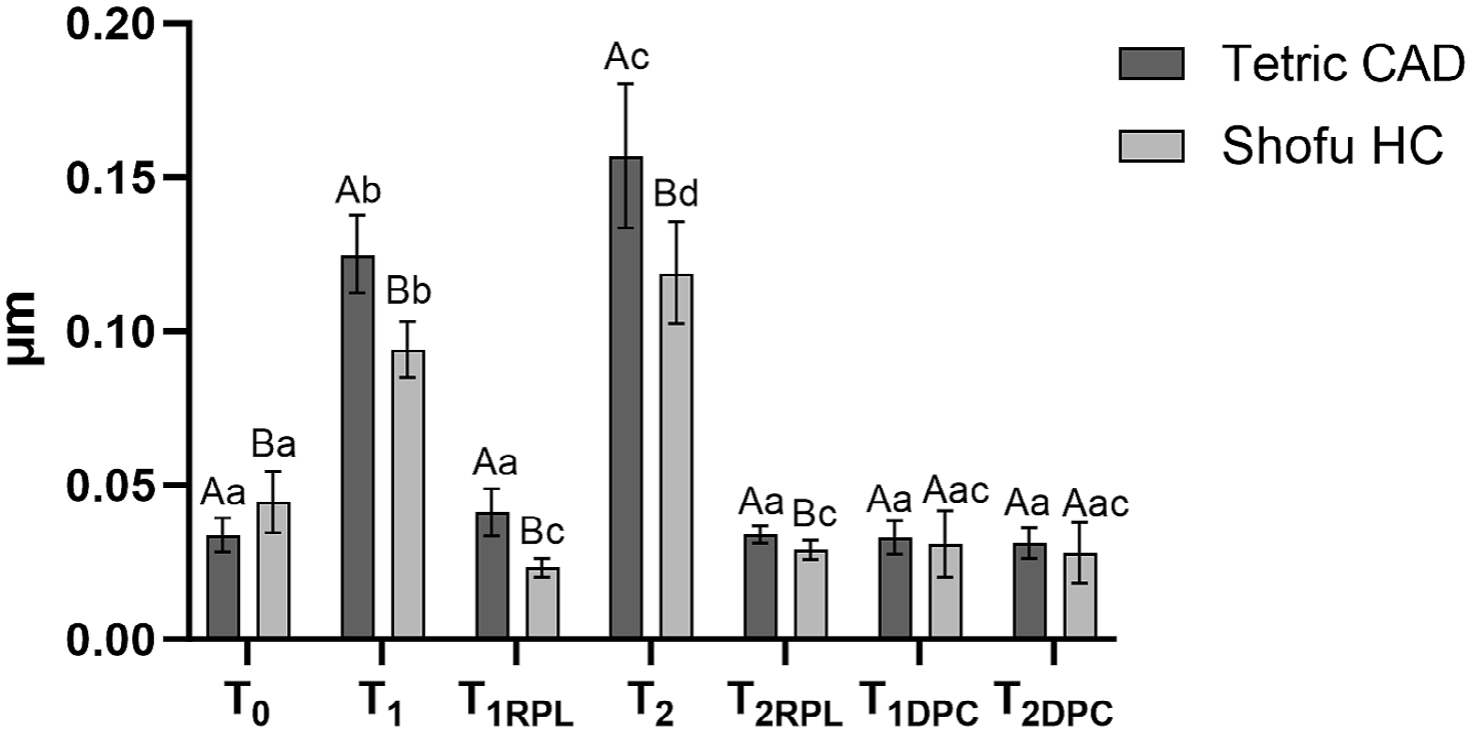
Mean values, standard deviations, and Tukey post hoc comparisons for surface roughness according to the evaluation periods, including the additional double‐polished control groups (T_1DPC_ and T_2DPC_). Uppercase letters indicate differences between the different materials within the same evaluation period, while lowercase letters indicate differences between the different evaluation periods for the same material.

Regarding Vickers microhardness, both materials showed lower values at T_2_ than at all other evaluation periods (*p* < 0.05). For TC, VH values at T_1_ and T_1 RPL_ were similar to those observed at the initial period (T_0_) (*p* = 1.000 and 0.939, respectively). Additionally, the group subjected to the longest aging period with periodic repolishing (T_2RPL_) showed significantly higher microhardness than its non‐repolished counterpart (T_2_). For SH, T_1RPL_ was the only group that did not differ from the initial period (T_0_) (*p* = 1.000). Both periodic repolished groups (T_1RPL_ and T_2RPL_) showed higher microhardness than their non‐repolished counterparts (T_1_ and T_2_) (*p* < 0.05). Furthermore, SH exhibited higher microhardness than TC at all evaluation periods (*p* < 0.05). The data and comparisons for Vickers microhardness are presented in Figure 4.

**FIGURE 4:**
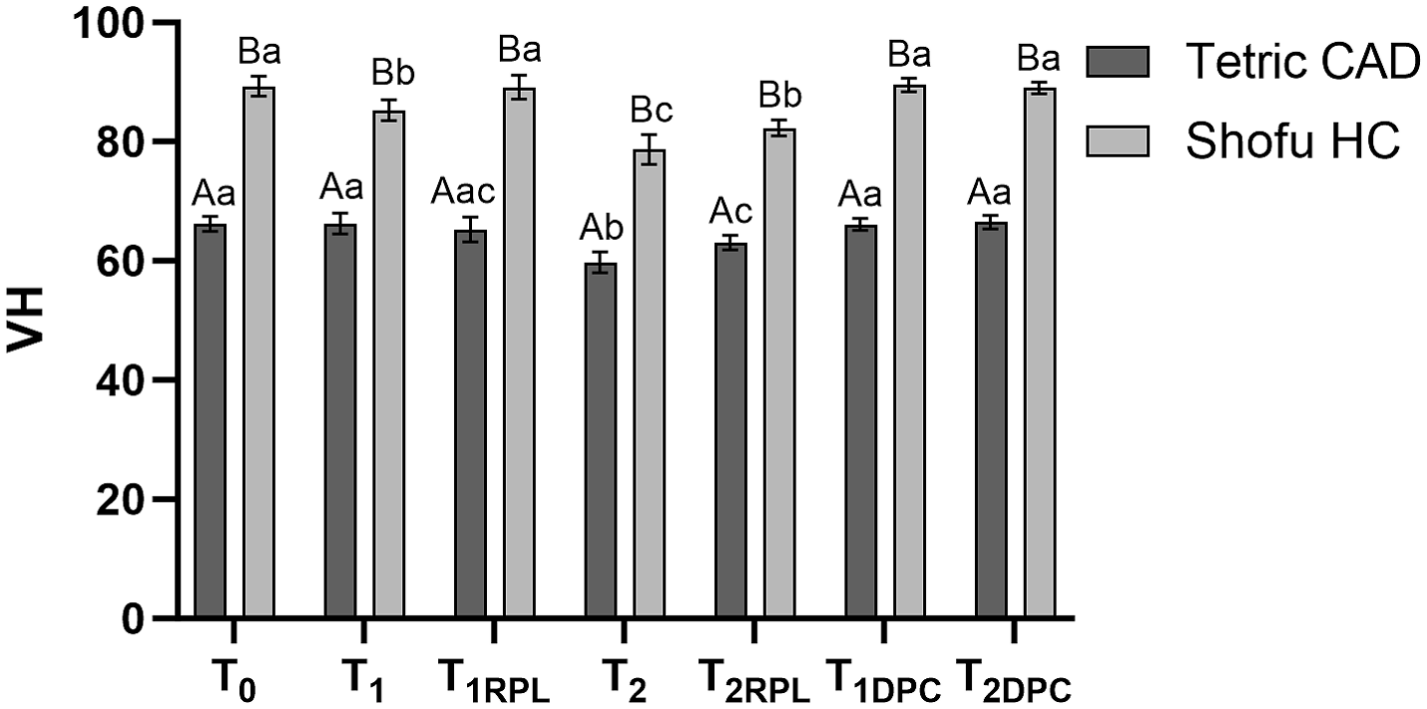
Mean values, standard deviations, and Tukey post hoc comparisons for Vickers microhardness according to the evaluation periods, including the additional double‐polished control groups (T_1DPC_ and T_2DPC_). Uppercase letters indicate differences between the different materials within the same evaluation period, while lowercase letters indicate differences between the different evaluation periods for the same material.

The additional double‐polished control groups (T_1DPC_ and T_2DPC_) were included in the surface roughness and Vickers microhardness analyses to assess the possible cumulative effect of repeated polishing. For surface roughness (Figure 3), DPC groups did not differ statistically from the initial condition (T_0_) or from the periodically repolished groups (T_1RPL_ and T_2RPL_) for both materials (TC: *p* = 1.000 and SH: *p* = 119). Similarly, for Vickers microhardness (Figure 4), DPC groups showed values comparable to those observed at T_0_, with no significant alteration after successive repolishing procedures without intervening aging (*p* = 1.000).

For flexural strength (Table 5), the analysis of variance did not indicate significant effects for the factors material (*p* = 0.783) and evaluation period (*p* = 0.646), nor for the interaction between them (*p* = 0.664). This indicates that no differences in flexural strength were observed between the evaluated materials or across the different evaluation periods. The flexural strength results are shown in Figure 5.

**TABLE 5:** Two‐way ANOVA results for flexural strength.

	Sum of squares	df	Mean square	*F*	*p*	*η* ^2^
Material	29.5	1	29.5	0.0762	0.783	0.001
Evaluation period	969.6	4	242.4	0.6249	0.646	0.036
Material × evaluation period	931.1	4	232.8	0.6001	0.664	0.035

**FIGURE 5:**
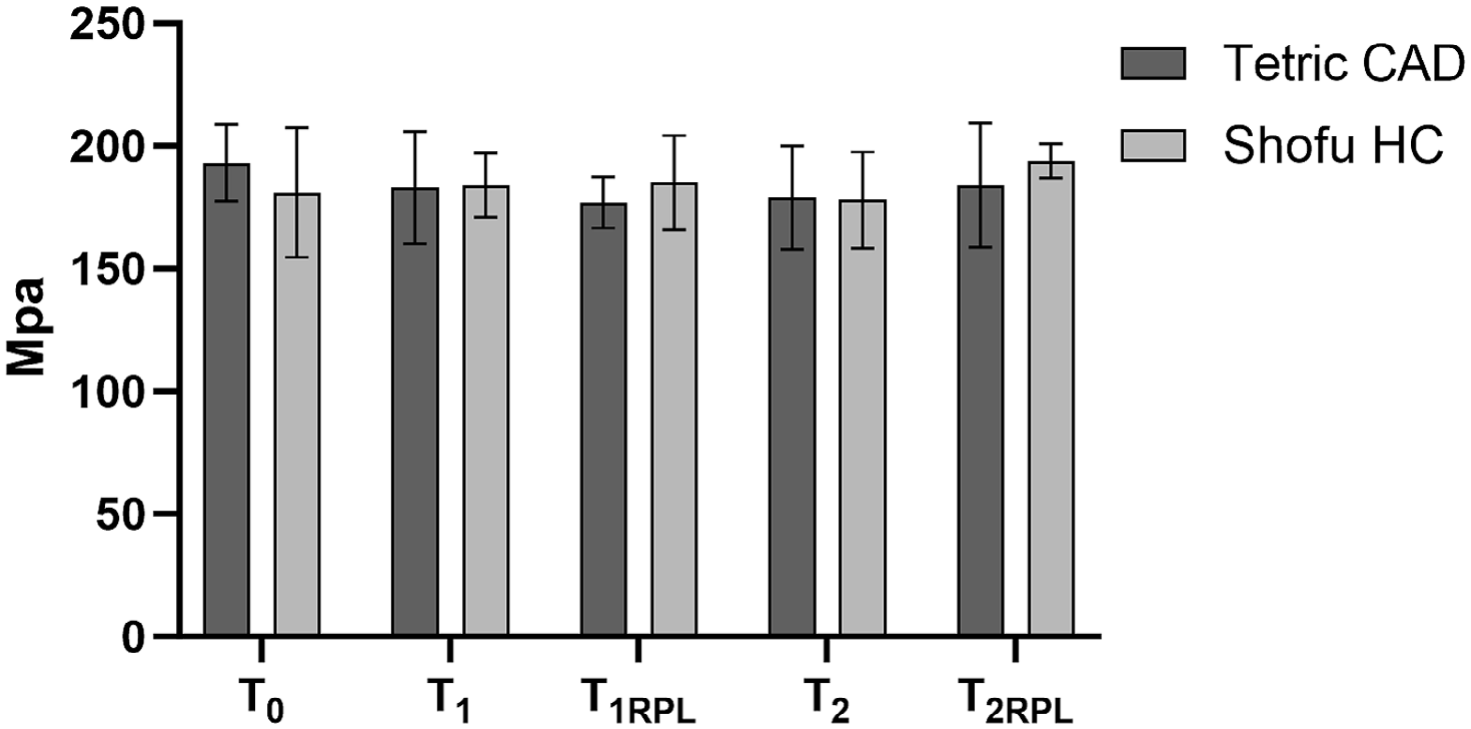
Mean values and standard deviations for flexural strength according to the evaluation periods.

Regarding elastic modulus (Table 6), the statistical analysis revealed a significant difference only for the material factor (*p* < 0.001), but not for the evaluation period (*p* = 0.342) or for the interaction between the two factors (*p* = 0.185). Therefore, differences in this property were observed among the tested materials, regardless of the evaluation period. Elastic modulus data are presented in Figure 6.

**TABLE 6:** Two‐way ANOVA results for elastic modulus.

	Sum of squares	df	Mean square	*F*	*p*	*η* ^2^
Material	4.04e+7	1	4.04e+7	53.77	< 0.001	0.453
Evaluation period	3.46e+6	4	865,816	1.15	0.342	0.039
Material × evaluation period	4.84e+6	4	1.21e+6	1.61	0.185	0.054

**FIGURE 6:**
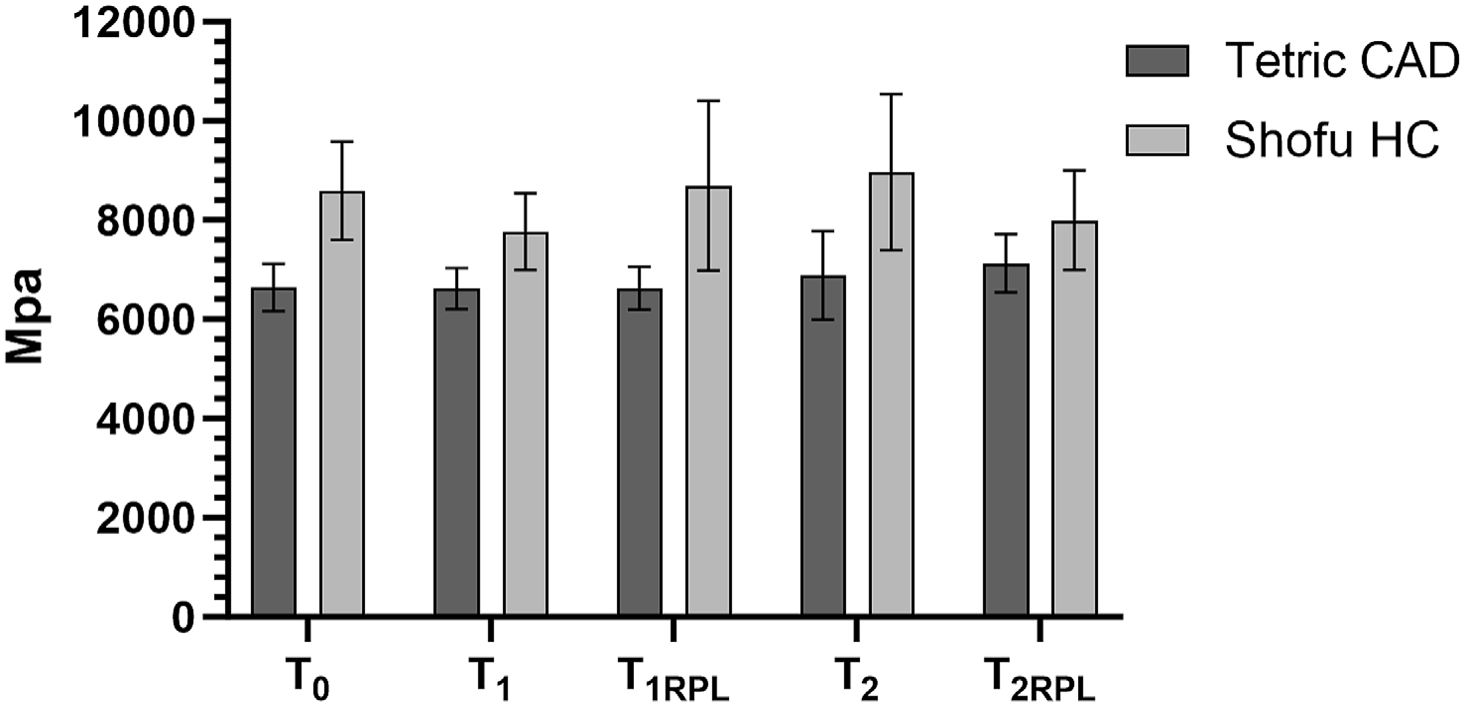
Mean values and standard deviations for elastic modulus according to the evaluation periods.

Since no significant differences in flexural strength or elastic modulus were observed among the non‐repolished groups throughout accelerated aging, it was expected that the periodically repolished groups would likewise show no significant changes.

SEM images showed that, at the initial period (Figures 7A and 8A), both materials exhibited a homogeneous surface morphology characterized by fine, oriented marks consistent with the abrasive movement produced during polishing. Filler particles appeared well integrated within the resin matrix, with no evident particle exposure or detachment. TC (Figure 7A) exhibited a more compact and uniform surface morphology.

**FIGURE 7:**
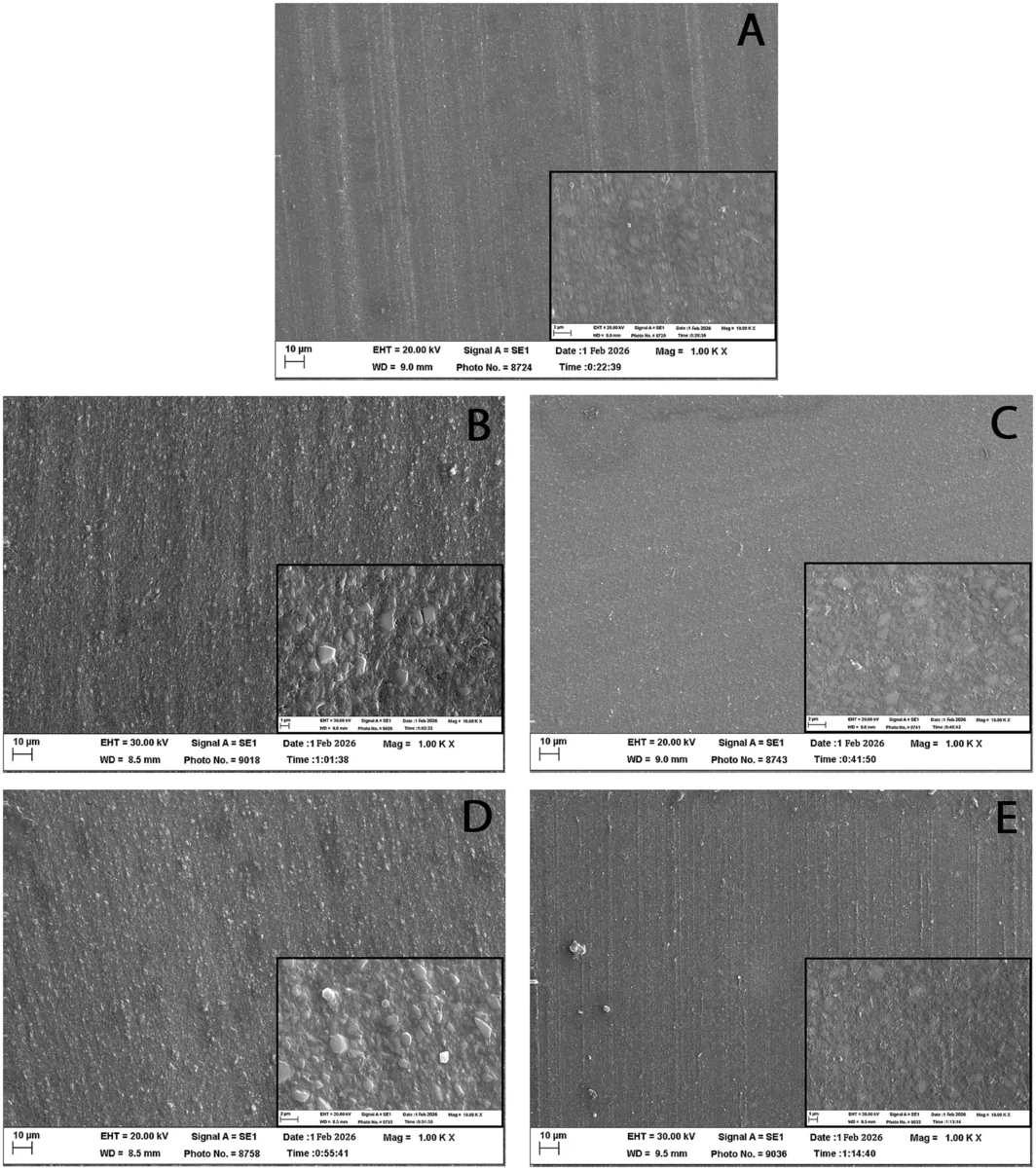
SEM micrographs (1000× and 10,000× magnification) of Tetric CAD surfaces at the different evaluation periods. (A) T_0_; (B) T_1_; (C) T_1RPL_; (D) T_2_; (E) T_2RPL_.

**FIGURE 8:**
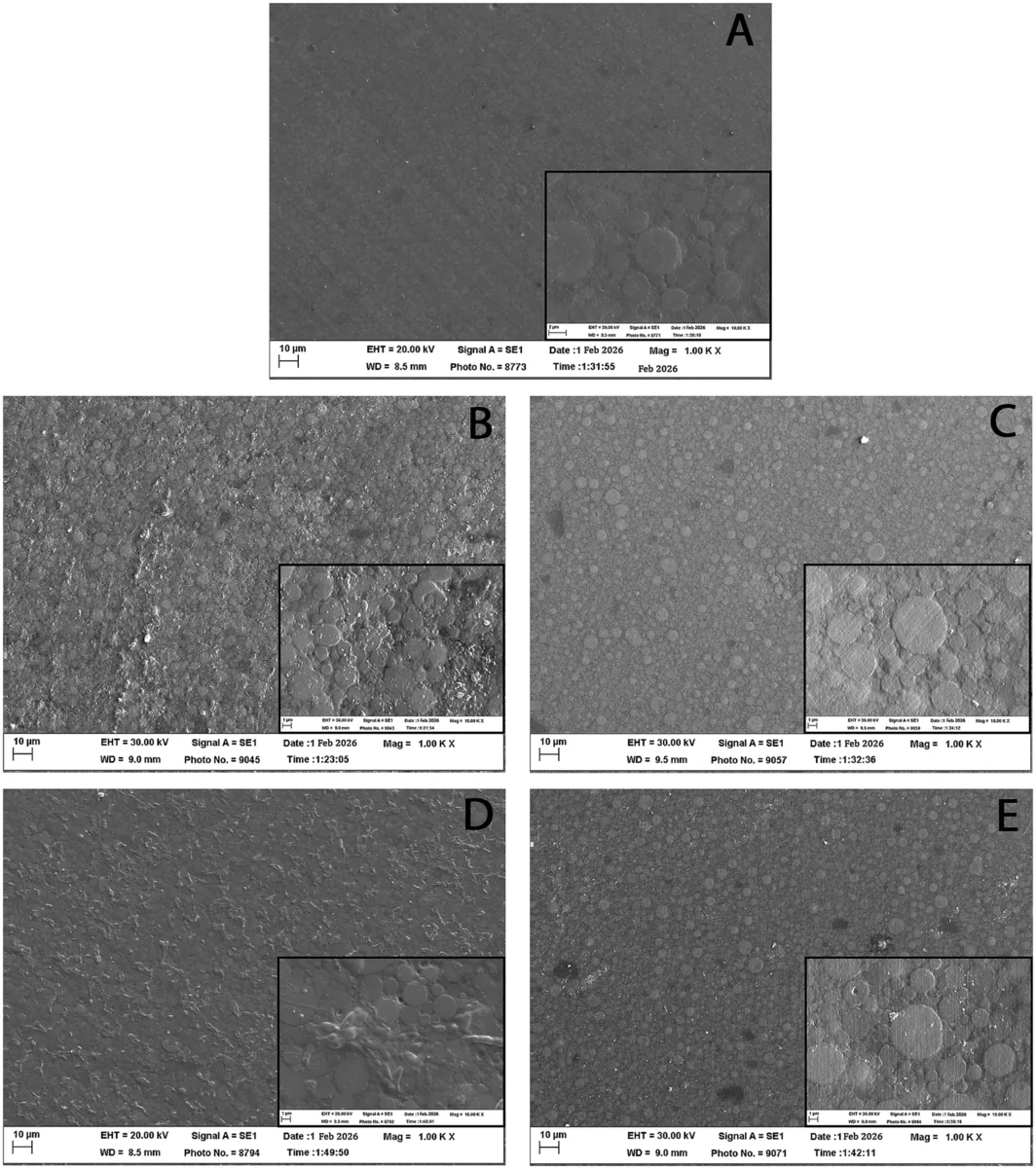
SEM micrographs (1000× and 10,000× magnification) of Shofu HC surfaces at the different evaluation periods. (A) T_0_; (B) T_1_; (C) T_1RPL_; (D) T_2_; (E) T_2RPL_.

After the aging periods without periodic repolishing (Figures 7B,D, 8B,D), the surfaces of both materials exhibited increased irregularities and signs of matrix degradation, including microdefects, deeper grooves, and surface discontinuities. For SH (Figure 8B,D), filler particle plucking was evident, characterized by microvoids and localized surface pits. For TC, the surface exhibited more generalized irregularities and a rougher topographic pattern (Figure 7B,D). In the groups subjected to periodic repolishing (Figures 7C,E and 8C,E), the surfaces showed a more homogeneous morphology, with fewer grooves, defects, and surface irregularities than the aged specimens without repolishing.

AFM images revealed that both materials exhibited a more homogeneous surface topography with lower irregularity amplitude at T_0_, T_1 RPL_, and T_2 RPL_ (Figures 9A,C,E and 10A,C,E). In contrast, at T_1_ and T_2_, the surfaces exhibited more pronounced peaks and valleys, reflecting increased topographic irregularities (Figures 9B,D and 10B,D).

**FIGURE 9:**
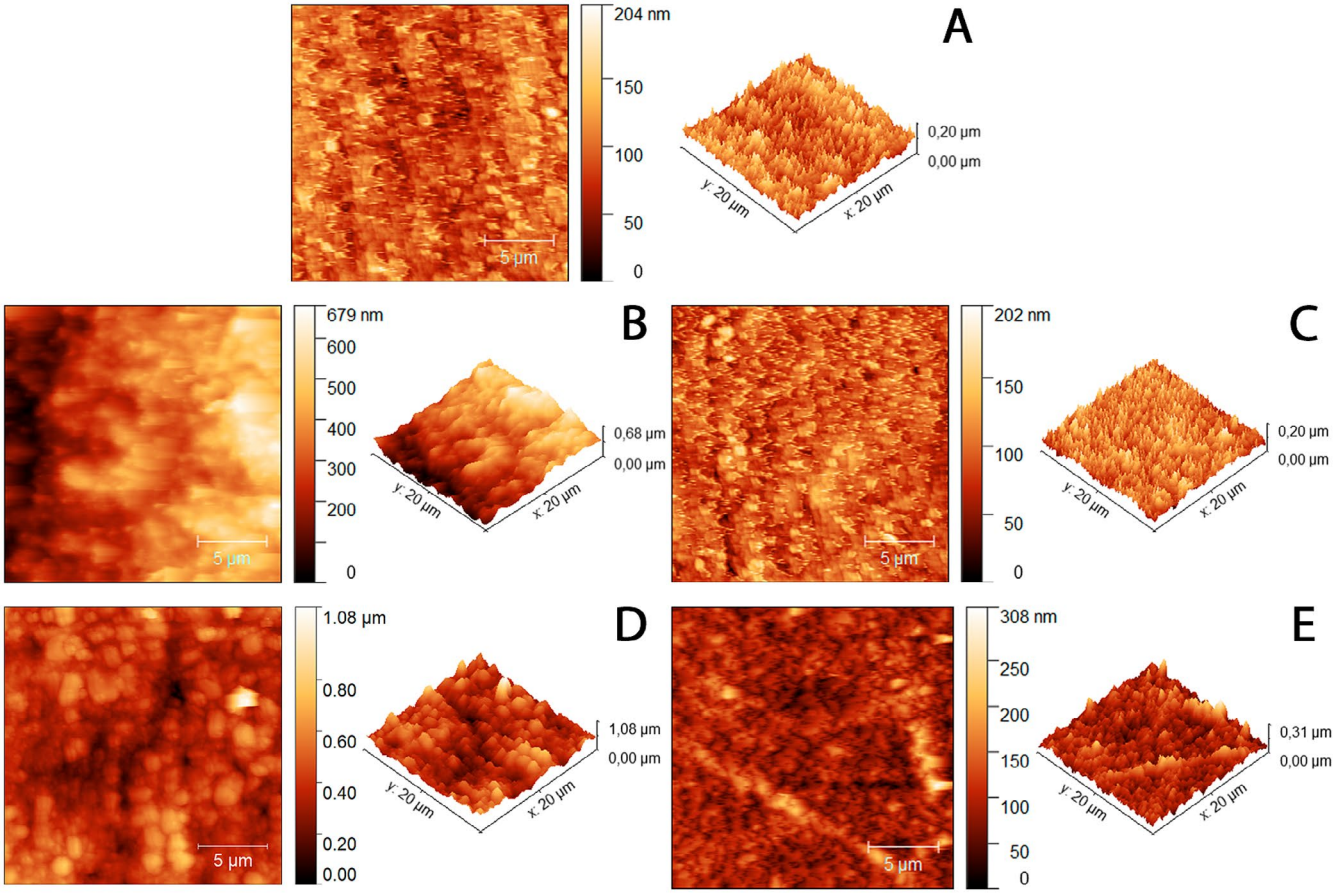
AFM images (20 × 20 μm) of Tetric CAD specimens at the different evaluation periods (2D and 3D). (A) T_0_; (B) T_1_; (C) T_1RPL_; (D) T_2_; (E) T_*2*RPL_.

**FIGURE 10:**
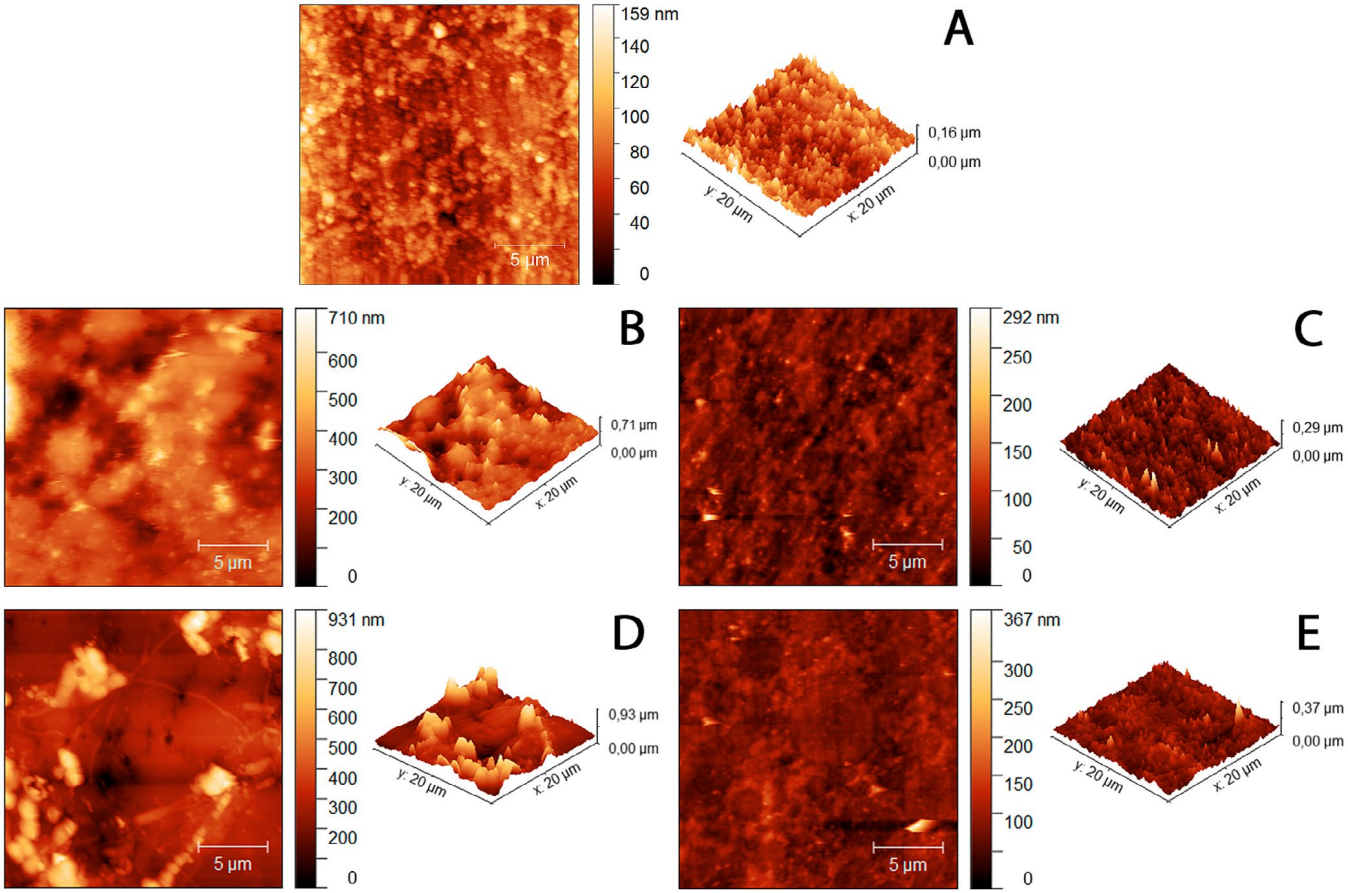
AFM images (20 × 20 μm) of Shofu HC specimens at the different evaluation periods (2D and 3D). (A) T_0_; (B) T_1_; (C) T_1RPL_; (D) T_2_; (E) T_2RPL_.

These morphological and topographical findings were consistent with the surface roughness results observed for the corresponding experimental conditions.

## 4 Discussion

The research hypothesis that periodic repolishing would influence the surface and mechanical properties of aged resin‐based CAD/CAM materials was partially accepted, since differences between repolished and non‐repolished groups were observed for surface roughness and microhardness.

Mechanical polishing enables the controlled removal of surface irregularities, promoting a more homogeneous exposure of the material microstructure and consequently reducing areas more susceptible to wear and degradation [46, 48, 49, 50, 51]. In this context, surface roughness appeared particularly sensitive to the combined effects of mechanical cycling and simulated toothbrushing. In the non‐repolished groups, a progressive increase in Ra was observed throughout the aging periods, reinforcing that even conditions considered clinically acceptable may result in progressive degradation of the restorative surface over time. Abrasive wear is characterized by material removal and may occur either through the direct interaction of a hard surface or asperity sliding against another surface or through hard abrasive particles interposed between moving surfaces [52]. In the present study, particles of the abrasive agent (hydrated silica) contained in the dentifrice were interposed between the toothbrush bristles and the material surfaces during the simulated brushing. The use of a medium‐abrasive dentifrice (Relative Dentin Abrasiveness of 70/78) was intentional, as it represents a realistic clinical condition and is widely recommended for the hygiene of direct and indirect composite resin restorations, justifying its selection for the present experiment [29, 53].

Although subjected to the same initial surface treatment, significant differences in Ra values were observed between the materials before and after aging. Statistical analysis showed higher Ra values for SH than TC only at T_0_ (*p* < 0.05), whereas lower values were observed for SH at all other evaluation periods (*p* < 0.05). These differences may be attributed to compositional characteristics, since the evaluated materials differ in the type, shape, size, distribution, and orientation of the inorganic fillers, factors that directly influence polishing quality and wear resistance [46, 50]. Shofu HC is classified as a microhybrid material containing approximately 61% inorganic particles with a more regular shape (Figure 8) and average particle sizes ranging from 1 to 10 μm, which may increase the difficulty of achieving a uniformly leveled surface. In contrast, Tetric CAD is a nanohybrid material containing 71% inorganic filler particles smaller than 1 μm (Figure 7), favoring the polishing procedure and the attainment of a smoother surface. It is worth noting that although manufacturers recommend specific polishing systems, individual clinician choices may also perform adequately with these materials.

Despite presenting lower initial roughness values, Tetric CAD exhibited a greater increase in surface roughness after aging, indicating higher susceptibility to surface degradation. While the initial roughness is primarily influenced by filler size, the progression of surface degradation over time appears to be more closely related to wear resistance and matrix‐related properties. Shofu HC contains inorganic particles with higher hardness, such as silica and zirconium silicate, incorporated into an organic matrix composed of UDMA and TEGDMA. This composition may provide greater resistance than the barium aluminum silicate inorganic fillers and the Bis‐GMA‐based organic matrix present in Tetric CAD due to the higher concentration of double bonds and the greater degree of crosslinking [46, 50]. The higher inorganic filler content in Tetric CAD cannot be considered a predictor of greater wear resistance. Previous studies have shown that these parameters do not exhibit a linear correlation and that the roughness behavior of these materials is more closely related to filler characteristics such as size, distribution, and type [14, 29, 50]. The smaller increase in surface roughness observed for Shofu HC may also be attributed to the quality of the matrix‐filler interface, with stronger interfacial bonding reducing the detachment of inorganic particles from the surface. In addition, filler agglomerates may become exposed due to abrasive action without being completely removed, limiting organic matrix exposure and contributing to the preservation of surface integrity [27, 50, 54, 55].

While the non‐repolished groups showed significant increases in surface roughness throughout the aging periods, the periodically repolished groups restored values similar to or even lower than those observed at the initial condition. This finding demonstrates that repolishing is capable of reversing accumulated surface degradation by promoting the controlled removal of irregularities formed during clinical use and restoring a more homogeneous surface topography. Such surface smoothing may help reduce biofilm accumulation and the development of secondary caries and periodontal disease. In addition, a more uniform surface reduces superficial defects that could propagate during functional loading [19, 22, 48, 49]. This understanding is particularly relevant, since periodic repolishing has been scarcely investigated as a systematic maintenance strategy for indirect restorations made of resin‐based materials. These effects were also observed in the SEM and AFM images, which show a marked reduction in surface irregularities after repolishing, as well as less pronounced peaks and valleys (Figures 7C,E, 8C,E, 9C,E, 10C,E).

A similar behavior was observed for microhardness. Although intrinsic differences between the materials explain the distinct absolute values, aging promoted a reduction in microhardness, particularly in the non‐repolished groups. Statistical analysis demonstrated that Shofu HC exhibited higher microhardness than Tetric CAD at all evaluation periods (*p* < 0.05). This finding highlights the superior intrinsic hardness of this material, which is associated with the hardness of its filler particles and their greater resistance to indentation during testing.

Regarding microhardness stability throughout the experiment, TC exhibited greater stability, with significant reductions (*p* < 0.05) observed only at T_2_ and T_2 RPL_ compared with T_0_. In contrast, SH showed significant reductions (*p* < 0.05) from the first evaluation period (T_1_), with the lowest value recorded at T_2_ (*p* < 0.05). This behavior may be associated with the higher proportion of organic matrix present in SH. During the mechanical cycling test, specimens remained immersed in distilled water, while water was also present in the dentifrice slurry during simulated brushing. Direct contact with an aqueous environment may promote water diffusion into the resin matrix, which acts as a plasticizing agent [56, 57, 58, 59]. As the organic matrix volume fraction is greater in SH, a larger volume is available for water diffusion, resulting in increased plasticization and consequently reduced microhardness [57, 58, 59]. These findings suggest that the observed reduction in microhardness cannot be attributed to a single factor, but rather to the combined effect of water exposure and mechanical aging. Although differences in polymer matrix composition may influence water affinity, filler content appears to play a more relevant role in this behavior [57, 58, 59]. Materials with similar monomer compositions may exhibit different water sorption, whereas distinct compositions may show comparable behavior, indicating that the matrix‐to‐filler ratio is a key determinant [57, 60, 61]. In addition, higher filler content has been associated with reduced water diffusion and smaller decreases in microhardness over time [57, 58, 59], which may explain the greater stability observed for TC.

Similarly to surface roughness, periodic repolishing proved effective in improving microhardness stability over time. This effect may be associated with the reconditioning of the superficially degraded layer, thereby exposing a less affected subsurface region of the material [30, 62, 63, 64, 65]. Previous studies have reported increases in surface microhardness following repolishing procedures, as well as the removal of superficial alterations such as staining and degradation [30, 62, 63, 64, 65]. These findings support the interpretation that aging‐induced surface changes may be, at least partially, reversible through repolishing. For SH, repolishing at the first evaluation period (T_1_) resulted in microhardness values comparable to the initial condition (T_0_), whereas at the second period (T_2_) the repolished group showed values similar to those observed at T_1_. For TC, the repolished group at the second evaluation period (T_2 RPL_) exhibited significantly higher values than the non‐repolished group (*p* < 0.05). Overall, the repolished groups showed more favorable microhardness values than the non‐repolished groups for both materials. These findings are consistent with previous evidence suggesting that repolishing may contribute to the partial regaining of compromised microhardness in resin‐based materials, potentially reducing abrasive wear over time associated with clinical use, including toothbrushing and chewing [30].

The inclusion of the DPC groups strengthens these interpretations by addressing the possible confounding effect of repeated polishing itself. Because the DPC groups were subjected to successive repolishing procedures without intervening aging and showed surface roughness and Vickers microhardness values comparable to baseline, the more favorable results observed in the aged repolished groups cannot be attributed solely to a cumulative mechanical polishing effect. Instead, these findings suggest that periodic repolishing primarily acts by reconditioning the aging‐induced superficial alterations, likely through the removal or smoothing of the degraded outer layer and exposure of a less affected subsurface region. Therefore, the DPC findings support the interpretations that repolishing contributed to the recovery or preservation of surface‐related properties after aging, rather than simply improving the surface by repeated mechanical polishing alone.

Adequate flexural properties are essential for restorative materials to withstand functional loads and reduce the risk of fracture [57, 66, 67, 68]. Fatigue tests conducted in an aqueous environment are capable of adequately reproducing failure mechanisms associated with subcritical crack growth, a phenomenon commonly observed under clinical conditions. The combination of cyclic loading and water may promote the gradual propagation of cracks originating from preexisting structural defects in ceramic materials [69, 70].

Interestingly, despite prior mechanical cycling, no significant reductions in flexural strength or elastic modulus were observed, suggesting that the applied aging protocol was insufficient to induce structural damage capable of compromising fracture behavior. This finding may be explained by the retardation of crack propagation in resin‐based materials resulting from the presence of water within the organic matrix. The plasticizing effect reduces stress concentration at the crack tip and promotes energy dissipation [68, 69, 71, 72, 73, 74]. This behavior prevents localized surface alterations from evolving into structural failures. These results are consistent with previous evidence indicating that resin‐based materials may maintain stable or favorable mechanical behavior after aging or fatigue under specific testing conditions [43, 68, 69, 73, 74]. However, it should be considered that water may also contribute to hydrolytic degradation and that the increased surface roughness observed after aging could act as crack initiation sites. In the present study, although roughness values increased, they remained low, likely limiting their role as critical stress concentrators. In addition, the total water exposure time was relatively short, which may have restricted the extent of hydrolytic degradation. Grzebieluch et al. [59] reported that, after 3 months of water storage, the same materials evaluated in the present study showed an average reduction of approximately 17% in flexural strength [59]. In contrast, the total water exposure time in the present study did not exceed 14 days, representing a period approximately 85% shorter than that previously reported. Therefore, the absence of significant changes in flexural strength may be attributed to a balance between these competing mechanisms, combined with the inherent damage‐tolerant behavior of resin‐based materials [14, 38, 43, 57, 59, 68, 69, 73, 74].

Calculating elastic modulus improves understanding of material stiffness and behavior under load. The results showed that SH exhibited higher elastic modulus values than TC at all evaluation periods (*p* < 0.05). This finding diverges from studies that have associated increases in this property with the amount of inorganic filler present in the material and may also be related to other factors, such as filler composition and type, the efficiency of the filler‐matrix interface, and the structure of the polymer network formed during industrial block processing [17, 47, 75, 76].

Overall, the findings of the present study indicate that in vitro aging predominantly affects surface properties sensitive to the initial degradation of the resin matrix and filler–matrix interface, whereas bulk properties remain stable. In this context, periodic intraoral repolishing emerges as a simple, accessible, and effective clinical strategy capable of minimizing the deleterious effects of aging, preserving surface integrity, and potentially increasing the clinical longevity [77] of indirect restorations fabricated from resin‐based CAD CAM materials.

The present study presents limitations inherent to its in vitro design, as it is not possible to fully reproduce the complex conditions of the oral environment, including variations in pH, temperature, diet, and individual hygiene habits. In addition, only two resin‐based CAD CAM materials and a single clinical polishing system were evaluated, which may limit the extrapolation of the findings to other materials and polishing protocols. Future studies employing cross‐sectional analyses or depth‐profile mechanical characterization would be valuable for further elucidating the mechanisms involved in the effects of repolishing on surface microhardness. Finally, the equivalence between accelerated aging and real clinical time should be interpreted with caution.

## 5 Conclusion

In vitro aging by mechanical cycling combined with simulated toothbrushing significantly affected the surface roughness and microhardness of both resin‐based CAD/CAM materials, while flexural strength and elastic modulus remained unchanged. Periodic intraoral repolishing proved to be an effective strategy for minimizing the effects of aging, reducing surface roughness and preserving microhardness. Therefore, periodic repolishing may be considered a relevant clinical maintenance protocol for indirect restorations fabricated from resin‐based CAD/CAM materials, thereby preserving surface integrity.

## Funding

This work was supported by São Paulo Research Foundation (FAPESP) (grants no. 2024/13572‐7, 2021/08529‐7), Coordenação de Aperfeiçoamento de Pessoal de Nível Superior (CAPES) (Finance Code 001), and Conselho Nacional de Desenvolvimento Científico e Tecnológico (CNPq) (grants no. 406508/2023‐2, 426719/2018‐2).

## Conflicts of Interest

The authors declare no conflicts of interest.

## Supporting information


**Table S1:** Mean ± standard deviation values for all evaluated outcomes according to material and experimental group.

## Data Availability

The data that support the findings of this study are available from the corresponding author upon reasonable request.

## References

[1] P. M. Soares , A. M. O. Dal Piva , G. K. R. Pereira , et al., “Effect of Brushing Simulation on the Wear Behavior of Repaired CAD‐CAM Restorations,” International Dental Journal 74, no. 5 (2024): 999–1005, 10.1016/j.identj.2024.02.012. PMC11561493 38461097

[2] W. Grzebieluch , M. Mikulewicz , and U. Kaczmarek , “Resin Composite Materials for Chairside CAD/CAM Restorations: A Comparison of Selected Mechanical Properties,” Journal of Healthcare Engineering 2021 (2021): 8828954, 10.1155/2021/8828954. PMC8099508 34007429

[3] G. Çakmak , M. G. Subaşı , and B. Yilmaz , “Effect of Thermocycling on the Surface Properties of Resin‐Matrix CAD‐CAM Ceramics After Different Surface Treatments,” Journal of the Mechanical Behavior of Biomedical Materials 117 (2021): 104401, 10.1016/j.jmbbm.2021.104401. 33652235

[4] M. I. Elmokadem , K. M. Haggag , and H. R. Mohamed , “Effect of Thermo‐Mechanical Cycling on Fracture Resistance of Different CAD/CAM Crowns: An In Vitro Study,” Journal of Contemporary Dental Practice 25, no. 1 (2024): 29–34, 10.5005/jp-journals-10024-3619. 38514428

[5] N. Attik , P. Colon , B. Grosgogeat , F. Z. Cherchali , and H. Abouelleil , “Machinability and Mechanical Properties of Four CAD/CAM Block Materials,” European Journal of Oral Sciences 133, no. 5 (2025): e70030, 10.1111/eos.70030. 40641040

[6] F. A. Shembish , H. Tong , M. Kaizer , et al., “Fatigue Resistance of CAD/CAM Resin Composite Molar Crowns,” Dental Materials 32, no. 4 (2016): 499–509, 10.1016/j.dental.2015.12.005. PMC4801772 26777092

[7] R. A. Alamoush , N. Silikas , N. A. Salim , S. Al‐Nasrawi , and J. D. Satterthwaite , “Effect of the Composition of CAD/CAM Composite Blocks on Mechanical Properties,” BioMed Research International 2018 (2018): 4893143, 10.1155/2018/4893143. PMC6218798 30426009

[8] R. A. Alamoush , J. D. Satterthwaite , N. Silikas , and D. C. Watts , “Viscoelastic Stability of Pre‐Cured Resin‐Composite CAD/CAM Structures,” Dental Materials 35, no. 8 (2019): 1166–1172, 10.1016/j.dental.2019.05.007. 31146959

[9] L. H. Schlichting , H. P. Maia , L. N. Baratieri , and P. Magne , “Novel‐Design Ultra‐Thin CAD/CAM Composite Resin and Ceramic Occlusal Veneers for the Treatment of Severe Dental Erosion,” Journal of Prosthetic Dentistry 105, no. 4 (2011): 217–226, 10.1016/S0022-3913(11)60035-8. 21458646

[10] J. Sripetchdanond and C. Leevailoj , “Wear of Human Enamel Opposing Monolithic Zirconia, Glass Ceramic, and Composite Resin: An In Vitro Study,” Journal of Prosthetic Dentistry 112, no. 5 (2014): 1141–1150, 10.1016/j.prosdent.2014.05.006. 24980740

[11] F. A. Spitznagel , K. J. Scholz , J. R. Strub , K. Vach , and P. C. Gierthmuehlen , “Polymer‐Infiltrated Ceramic CAD/CAM Inlays and Partial Coverage Restorations: 3‐Year Results of a Prospective Clinical Study Over 5 Years,” Clinical Oral Investigations 22, no. 5 (2018): 1973–1983, 10.1007/s00784-017-2293-x. 29214376

[12] G. D. Siddanna , A. J. Valcanaia , P. H. Fierro , G. F. Neiva , and D. J. Fasbinder , “Surface Evaluation of Resilient CAD/CAM Ceramics After Contouring and Polishing,” Journal of Esthetic and Restorative Dentistry 33, no. 5 (2021): 750–763, 10.1111/jerd.12735. 33973352

[13] T. S. Porto , R. C. Roperto , A. Akkus , et al., “Effect of Storage and Aging Conditions on the Flexural Strength and Flexural Modulus of CAD/CAM Materials,” Dental Materials Journal 38, no. 2 (2019): 264–270, 10.4012/dmj.2018-111. 30541991

[14] N. C. Lawson , R. Bansal , and J. O. Burgess , “Wear, Strength, Modulus and Hardness of CAD/CAM Restorative Materials,” Dental Materials 32, no. 11 (2016): e275–e283, 10.1016/j.dental.2016.08.222. 27639808

[15] N. D. Ruse and M. J. Sadoun , “Resin‐Composite Blocks for Dental CAD/CAM Applications,” Journal of Dental Research 93, no. 12 (2014): 1232–1234, 10.1177/0022034514553976. PMC4462808 25344335

[16] B. Hussain , M. K. L. Thieu , G. F. Johnsen , J. E. Reseland , and H. J. Haugen , “Can CAD/CAM Resin Blocks Be Considered as Substitute for Conventional Resins?,” Dental Materials 33, no. 12 (2017): 1362–1370, 10.1016/j.dental.2017.09.003. 28951036

[17] I. Papathanasiou , S. Zinelis , G. Papavasiliou , and P. Kamposiora , “Effect of Aging on Color, Gloss and Surface Roughness of CAD/CAM Composite Materials,” Journal of Dentistry 130 (2023): 104423, 10.1016/j.jdent.2023.104423. 36642325

[18] M. Tavangar , R. Bagheri , T. Y. Kwon , A. Mese , and D. J. Manton , “Influence of Beverages and Surface Roughness on the Color Change of Resin Composites,” Journal of Investigative and Clinical Dentistry 9, no. 3 (2018): e12333, 10.1111/jicd.12333. 29575793

[19] U. Lohbauer , F. A. Müller , and A. Petschelt , “Influence of Surface Roughness on Mechanical Strength of Resin Composite Versus Glass Ceramic Materials,” Dental Materials 24, no. 2 (2008): 250–256, 10.1016/j.dental.2007.05.006. 17628659

[20] N. Ilie , “Altering of Optical and Mechanical Properties in High‐Translucent CAD‐CAM Resin Composites During Aging,” Journal of Dentistry 85 (2019): 64–72, 10.1016/j.jdent.2019.05.015. 31078575

[21] K. J. Anusavice , K. Kakar , and N. Ferree , “Which Mechanical and Physical Testing Methods Are Relevant for Predicting the Clinical Performance of Ceramic‐Based Dental Prostheses?,” Clinical Oral Implants Research 18, no. S3 (2007): 218–231, 10.1111/j.1600-0501.2007.01460.x. 17594384

[22] N. Jager , A. J. Feilzer , and C. L. Davidson , “The Influence of Surface Roughness on Porcelain Strength,” Dental Materials 16, no. 6 (2000): 381–388, 10.1016/s0109-5641(00)00030-0. 10967186

[23] C. M. Bollen , P. Lambrechts , and M. Quirynen , “Comparison of Surface Roughness of Oral Hard Materials to the Threshold Surface Roughness for Bacterial Plaque Retention: A Review of the Literature,” Dental Materials 13, no. 4 (1997): 258–269, 10.1016/s0109-5641(97)80038-3. 11696906

[24] V. Preis , M. Behr , G. Handel , S. Schneider‐Feyrer , S. Hahnel , and M. Rosentritt , “Wear Performance of Dental Ceramics After Grinding and Polishing Treatments,” Journal of the Mechanical Behavior of Biomedical Materials 10 (2012): 13–22, 10.1016/j.jmbbm.2012.03.002. 22520415

[25] S. D. Heintze , A. Cavalleri , M. Forjanic , G. Zellweger , and V. Rousson , “Wear of Ceramic and Antagonist: A Systematic Evaluation of Influencing Factors In Vitro,” Dental Materials 24, no. 4 (2008): 433–449, 10.1016/j.dental.2007.06.016. 17720238

[26] O. Sagsoz , T. Demirci , G. Demirci , N. P. Sagsoz , and M. Yildiz , “The Effects of Different Polishing Techniques on the Staining Resistance of CAD/CAM Resin‐Ceramics,” Journal of Advanced Prosthodontics 8, no. 6 (2016): 417–422, 10.4047/jap.2016.8.6.417. PMC5179479 28018558

[27] S. Flury , E. Diebold , A. Peutzfeldt , and A. Lussi , “Effect of Artificial Toothbrushing and Water Storage on the Surface Roughness and Micromechanical Properties of Tooth‐Colored CAD‐CAM Materials,” Journal of Prosthetic Dentistry 117, no. 6 (2017): 767–774, 10.1016/j.prosdent.2016.08.034. 27836147

[28] S. G. Batmaz , S. N. Karakaş , and C. Küden , “Surface Characteristics and Bacterial Adhesion on Single‐Shade Composite Resins,” Dental Materials Journal 43, no. 6 (2024): 850–860, 10.4012/dmj.2024-041. 39462609

[29] G. S. Andrade , M. G. Augusto , B. V. Simões , C. Pagani , G. S. F. A. Saavedra , and E. Bresciani , “Impact of Simulated Toothbrushing on Surface Properties of Chairside CAD‐CAM Materials: An In Vitro Study,” Journal of Prosthetic Dentistry 125, no. 3 (2021): 469.e1–469.e6, 10.1016/j.prosdent.2020.08.028. 33279154

[30] H. Yang , R. Chen , T. Attin , H. Cheng , and H. Yu , “Repolishing In Situ Eroded CAD/CAM Restorative Materials and Human Enamel,” Journal of the Mechanical Behavior of Biomedical Materials 113 (2021): 104125, 10.1016/j.jmbbm.2020.104125. 33068923

[31] A. M. O. Dal Piva , M. A. Bottino , L. C. Anami , et al., “Toothbrushing Wear Resistance of Stained CAD/CAM Ceramics,” Coatings 11, no. 2 (2021): 224, 10.3390/coatings11020224.

[32] S. Ardu , V. Braut , I. Uhac , N. Benbachir , A. J. Feilzer , and I. Krejci , “Influence of Mechanical and Chemical Degradation on Surface Gloss of Resin Composite Materials,” American Journal of Dentistry 22, no. 5 (2009): 264–268. 20225467

[33] J. P. M. Tribst , A. M. O. Dal Piva , A. Werner , et al., “Effect of Surface Treatment and Glaze Application on Shade Characterized Resin‐Modified Ceramic After Toothbrushing,” Journal of Prosthetic Dentistry 125, no. 4 (2021): 691.e1–691.e7, 10.1016/j.prosdent.2020.12.040. 33820617

[34] F. R. Zanin , L. F. Garcia , L. A. Casemiro , and F. C. Pires‐de‐Souza , “Effect of Artificial Accelerated Aging on Color Stability and Surface Roughness of Indirect Composites,” European Journal of Prosthodontics and Restorative Dentistry 16, no. 1 (2008): 10–14. 18468319

[35] J. Costa , A. Adams‐Belusko , K. Riley , and J. L. Ferracane , “The Effect of Various Dentifrices on Surface Roughness and Gloss of Resin Composites,” Journal of Dentistry 38, no. Suppl 2 (2010): e123–e128, 10.1016/j.jdent.2010.02.005. 20193728

[36] L. M. Roselino , M. A. Chinelatti , C. C. Alandia‐Román , and F. C. Pires‐de‐Souza , “Effect of Brushing Time and Dentifrice Abrasiveness on Color Change and Surface Roughness of Resin Composites,” Brazilian Dental Journal 26, no. 5 (2015): 507–513, 10.1590/0103-6440201300399. 26647937

[37] L. Wang , F. C. Garcia , P. A. Araújo , E. B. Franco , and R. F. Mondelli , “Wear Resistance of Packable Resin Composites After Simulated Toothbrushing Test,” Journal of Esthetic and Restorative Dentistry 16, no. 5 (2004): 303–315, 10.1111/j.1708-8240.2004.tb00058.x. 15726799

[38] M. N. Aboushelib and M. H. Elsafi , “Survival of Resin Infiltrated Ceramics Under Influence of Fatigue,” Dental Materials 32, no. 4 (2016): 529–534, 10.1016/j.dental.2015.12.001. 26764178

[39] E. G. Mota , L. N. Smidt , L. M. Fracasso , L. H. Burnett, Jr. , and A. M. Spohr , “The Effect of Milling and Post Milling Procedures on the Surface Roughness of CAD/CAM Materials,” Journal of Esthetic and Restorative Dentistry 29, no. 6 (2017): 450–458, 10.1111/jerd.12326. 28891600

[40] N. E. Ozer and E. I. Oguz , “Influence of Different Finishing‐Polishing Procedures and Thermocycle Aging on the Surface Roughness of Nano‐Ceramic Hybrid CAD/CAM Material,” Nigerian Journal of Clinical Practice 26, no. 5 (2023): 604–611, 10.4103/njcp.njcp_592_22. 37357477

[41] R. Albani , S. R. Habib , A. AlQahtani , A. A. AlHelal , M. Alrabiah , and S. Anwar , “The Surface Roughness of Contemporary Indirect CAD/CAM Restorative Materials That Are Glazed and Chair‐Side Finished/Polished,” Materials 17, no. 5 (2024): 997, 10.3390/ma17050997. PMC10934925 38473470

[42] M. Matzinger , S. Hahnel , V. Preis , and M. Rosentritt , “Polishing Effects and Wear Performance of Chairside CAD/CAM Materials,” Clinical Oral Investigations 23, no. 2 (2019): 725–737, 10.1007/s00784-018-2473-3. 29770877

[43] S. H. Kim , Y. S. Choi , K. H. Kang , and W. Att , “Effects of Thermal and Mechanical Cycling on the Mechanical Strength and Surface Properties of Dental CAD‐CAM Restorative Materials,” Journal of Prosthetic Dentistry 128, no. 1 (2022): 79–88, 10.1016/j.prosdent.2020.11.014. 33546857

[44] M. Z. D. Picolo , M. Kury , D. Romário‐Silva , et al., “Effects of Gastric Acid and Mechanical Toothbrushing in CAD‐CAM Restorative Materials: Mechanical Properties, Surface Topography, and Biofilm Adhesion,” Journal of the Mechanical Behavior of Biomedical Materials 138 (2023): 105606, 10.1016/j.jmbbm.2022.105606. 36525873

[45] J. C. Yuan , V. A. R. Barão , A. G. Wee , M. F. Alfaro , F. S. Afshari , and C. Sukotjo , “Effect of Brushing and Thermocycling on the Shade and Surface Roughness of CAD‐CAM Ceramic Restorations,” Journal of Prosthetic Dentistry 119, no. 6 (2018): 1000–1006, 10.1016/j.prosdent.2017.06.001. 28965682

[46] M. Özarslan , D. Bilgili Can , N. H. Avcioglu , and S. Çalışkan , “Effect of Different Polishing Techniques on Surface Properties and Bacterial Adhesion on Resin‐Ceramic CAD/CAM Materials,” Clinical Oral Investigations 26, no. 8 (2022): 5289–5299, 10.1007/s00784-022-04497-8. 35474553

[47] P. V. Bora , A. Sayed Ahmed , A. Alford , K. Pittman , V. Thomas , and N. C. Lawson , “Characterization of Materials Used for 3D Printing Dental Crowns and Hybrid Prostheses,” Journal of Esthetic and Restorative Dentistry 36, no. 1 (2024): 220–230, 10.1111/jerd.13174. 38008797

[48] M. B. Schmidt , S. Hahnel , A. Rauch , and M. Rosentritt , “Influence of Surface Treatment on Roughness, Fracture Force, Flexural Strength, and Dynamic Loading of a 3D‐Printed Crown and Bridge Material,” Clinical Oral Investigations 29 (2025): 426, 10.1007/s00784-025-06518-8. PMC12398470 40884683

[49] U. Lohbauer , R. Belli , and J. L. Ferracane , “Factors Involved in Mechanical Fatigue Degradation of Dental Resin Composites,” Journal of Dental Research 92, no. 7 (2013): 584–591, 10.1177/0022034513490734. 23694927

[50] E. Ximinis , D. Dionysopoulos , C. Papadopoulos , A. Tournavitis , A. Konstantinidis , and O. Naka , “Effect of Tooth Brushing Simulation on the Surface Properties of Various Resin‐Matrix CAD/CAM Ceramics,” Journal of Esthetic and Restorative Dentistry 35, no. 6 (2023): 937–946, 10.1111/jerd.13043. 37052301

[51] G. Colak and G. Katirci , “In Vitro Evaluation of the Effects of Whitening Toothpastes on the Color and Surface Roughness of Different Composite Resin Materials,” BMC Oral Health 23 (2023): 580, 10.1186/s12903-023-03277-4. PMC10440036 37598143

[52] ASTM International , ASTM G40: Standard Terminology Relating to Wear and Erosion (ASTM, 1987).

[53] International Organization for Standardization , ISO 11609: Dentistry—Dentifrices—Requirements, Test Methods and Marking (ISO, 2017).

[54] G. U. Oliveira , R. F. Mondelli , M. C. Rodrigues , E. B. Franco , S. K. Ishikiriama , and L. Wang , “Impact of Filler Size and Distribution on Roughness and Wear of Composite Resin After Simulated Toothbrushing,” Journal of Applied Oral Science 20, no. 5 (2012): 510–516, 10.1590/s1678-77572012000500003. PMC3881789 23138735

[55] D. Angerame and M. De Biasi , “Do Nanofilled/Nanohybrid Composites Allow for Better Clinical Performance of Direct Restorations Than Traditional Microhybrid Composites? A Systematic Review,” Operative Dentistry 43, no. 4 (2018): E191–E209, 10.2341/17-212-L. 29570022

[56] N. E. Nam , S. H. Shin , J. H. Lim , J. S. Shim , and J. E. Kim , “Effects of Artificial Tooth Brushing and Hydrothermal Aging on the Mechanical Properties and Color Stability of Dental 3D Printed and CAD/CAM Materials,” Materials 14, no. 20 (2021): 6207, 10.3390/ma14206207. PMC8540203 34683798

[57] M. Lukomska‐Szymanska , M. Radwanski , N. Kharouf , et al., “Evaluation of Physical‐Chemical Properties of Contemporary CAD/CAM Materials With Chromatic Transition “Multicolor”,” Materials 16, no. 11 (2023): 4189, 10.3390/ma16114189. PMC10254467 37297323

[58] R. A. Alamoush , R. Sung , J. D. Satterthwaite , and N. Silikas , “The Effect of Different Storage Media on the Monomer Elution and Hardness of CAD/CAM Composite Blocks,” Dental Materials 37, no. 8 (2021): 1202–1213, 10.1016/j.dental.2021.04.012. 33962792

[59] W. Grzebieluch , P. Kowalewski , M. Sopel , and M. Mikulewicz , “Influence of Artificial Aging on Mechanical Properties of Six Resin Composite Blocks for CAD/CAM Application,” Coatings 12, no. 6 (2022): 837, 10.3390/coatings12060837.

[60] F. Fuchs , J. Schmidtke , S. Hahnel , and A. Koenig , “The Influence of Different Storage Media on Vickers Hardness and Surface Roughness of CAD/CAM Resin Composites,” Journal of Materials Science. Materials in Medicine 34, no. 3 (2023): 13, 10.1007/s10856-023-06713-7. PMC10024666 36934208

[61] R. A. Alamoush , N. A. Salim , N. Silikas , and J. D. Satterthwaite , “Long‐Term Hydrolytic Stability of CAD/CAM Composite Blocks,” European Journal of Oral Sciences 130, no. 1 (2022): e12834, 10.1111/eos.12834. 34897842

[62] A. L. de Oliveira , A. C. Botta , J. A. Campos , and P. P. Garcia , “Effects of Immersion Media and Repolishing on Color Stability and Superficial Morphology of Nanofilled Composite Resin,” Microscopy and Microanalysis 20, no. 4 (2014): 1234–1239, 10.1017/S1431927614001299. 24892306

[63] T. E. Anfe , C. M. Agra , and G. F. Vieira , “Evaluation of the Possibility of Removing Staining by Repolishing Composite Resins Submitted to Artificial Aging,” Journal of Esthetic and Restorative Dentistry 23, no. 4 (2011): 260–267, 10.1111/j.1708-8240.2011.00435.x. 21806759

[64] G. Paolone , C. Mazzitelli , F. Boggio , et al., “Effect of Different Artificial Staining Procedures on the Color Stability and Translucency of a Nano‐Hybrid Resin‐Based Composite,” Materials (Basel) 16, no. 6 (2023): 2336, 10.3390/ma16062336. PMC10058860 36984216

[65] M. Ugurlu , “Effect of Repolishing on the Discoloration of Indirect Composite Block, Nanohybrid, and Microhybrid Resin Composites,” European Oral Research 56, no. 3 (2022): 158–163, 10.26650/eor.20221066456. PMC9826705 36660222

[66] S. M. Fouda , M. M. Gad , P. Ellakany , et al., “Flexural Properties, Impact Strength, and Hardness of Nanodiamond‐Modified PMMA Denture Base Resin,” International Journal of Biomaterials 2022 (2022): 6583084, 10.1155/2022/6583084. PMC9288300 35855810

[67] L. Calabrese , F. Fabiano , L. M. Bonaccorsi , V. Fabiano , and C. Borsellino , “Evaluation of the Clinical Impact of ISO 4049 in Comparison With Miniflexural Test on Mechanical Performances of Resin‐Based Composite,” International Journal of Biomaterials 2015 (2015): 149798, 10.1155/2015/149798. PMC4359833 25815011

[68] F. Egilmez , G. Ergun , I. Cekic‐Nagas , P. K. Vallittu , and L. V. J. Lassila , “Does Artificial Aging Affect Mechanical Properties of CAD/CAM Composite Materials?,” Journal of Prosthodontic Research 62, no. 1 (2018): 65–74, 10.1016/j.jpor.2017.06.001. 28647224

[69] Y. Karevan , M. Eldafrawy , R. Herman , C. Sanchez , M. Sadoun , and A. Mainjot , “Fatigue Behavior of CAD‐CAM Composites Versus Lithium Disilicate Glass‐Ceramic,” Dental Materials 41, no. 10 (2025): 1222–1230, 10.1016/j.dental.2025.07.004. 40651880

[70] N. Ilie , T. J. Hilton , S. D. Heintze , et al., “Academy of Dental Materials Guidance—Resin Composites: Part I—Mechanical Properties,” Dental Materials 33, no. 8 (2017): 880–894, 10.1016/j.dental.2017.04.013. 28577893

[71] F. Takeshige , Y. Kawakami , M. Hayashi , and S. Ebisu , “Fatigue Behavior of Resin Composites in Aqueous Environments,” Dental Materials 23, no. 7 (2007): 893–899, 10.1016/j.dental.2006.06.031. 17007919

[72] H. K. Tayeb , N. Silikas , A. J. Alhaddad , and J. Satterthwaite , “Flexural Strength and Hardness Analysis of 3D‐Printed vs. Milled Resin Composites Indicated for Definitive Crowns,” Journal of Functional Biomaterials 16, no. 12 (2025): 468, 10.3390/jfb16120468. PMC12734218 41440644

[73] A. Elraggal , R. R. Afifi , R. A. Alamoush , I. A. Raheem , and D. C. Watts , “Effect of Acidic Media on Flexural Strength and Fatigue of CAD‐CAM Dental Materials,” Dental Materials 39, no. 1 (2023): 57–69, 10.1016/j.dental.2022.11.019. 36496258

[74] A. B. Venturini , C. Prochnow , G. K. R. Pereira , R. D. Segala , C. J. Kleverlaan , and L. F. Valandro , “Fatigue Performance of Adhesively Cemented Glass‐, Hybrid‐ and Resin‐Ceramic Materials for CAD/CAM Monolithic Restorations,” Dental Materials 35, no. 4 (2019): 534–542, 10.1016/j.dental.2019.01.013. 30686711

[75] G. N. Sampaio , J. P. J. O. Limírio , J. M. L. Gomes , C. A. A. Lemos , A. A. Pesqueira , and E. P. Pellizzer , “Evaluation of Mechanical Properties of CAD‐CAM Composite Resins for Milled Versus 3D Printed Definitive Restorations: A Systematic Review and Meta‐Analysis,” Journal of Prosthetic Dentistry 135 (2025): 744–752, 10.1016/j.prosdent.2025.05.024. 40506304

[76] M. A. Hassan , S. Zinelis , M. Hersberger‐Zurfluh , and T. Eliades , “Creep, Hardness, and Elastic Modulus of Lingual Fixed Retainers Adhesives,” Materials 12, no. 4 (2019): 646, 10.3390/ma12040646. PMC6416616 30795503

[77] S. Duarte, Jr. and J. H. Phark , “Advances in Dental Restorations: A Comprehensive Review of Machinable and 3D‐Printed Ceramic‐Reinforced Composites,” Journal of Esthetic and Restorative Dentistry 37, no. 1 (2025): 257–276, 10.1111/jerd.13371. PMC11913211 39558703

