# Supplementary material for: Effectiveness of Periodic Repolishing on the Surface and Mechanical Properties of Resin‐Based CAD/CAM Materials After Different Cycles of Combined Simulated Toothbrushing and Mechanical Cycling
Source: J Esthet Restor Dent. 2026 Jul 19;38(11):2217–32. doi: 10.1111/jerd.70235 (PMC13680786; doi:10.1111/jerd.70235)
Supplement: Supplementary file 1 [file JERD-38-2217-s001.docx]

**Supplementary Table S1.** Mean ± standard deviation values for all evaluated outcomes according to material and experimental group.

| **Material** | **Outcome** | **T_0_** | **T_1_** | **T_1 RPL_** | **T_2_** | **T_2 RPL_** | **T_1 DPC_** | **T_2 DPC_** |
| --- | --- | --- | --- | --- | --- | --- | --- | --- |
| Tetric CAD | Ra (µm) | 0.0338 ± 0.00554 | 0.1250 ± 0.01260 | 0.0412 ± 0.00776 | 0.1570 ± 0.02340 | 0.0340 ± 0.00283 | 0.0330 ± 0.00535 | 0.0333 ± 0.00535 |
|  | VH | 66.2 ± 1.27 | 66.3 ± 1.75 | 65.3 ± 2.11 | 59.8 ± 1.73 | 63.1 ± 1.21 | 66.1 ± 0.989 | 66.5 ± 1.15 |
|  | FS (MPa) | 192.8 ± 15.6 | 182.7 ± 22.9 | 176.7 ± 10.4 | 178.5 ± 21.1 | 184.2 ± 25.3 | NE | NE |
|  | EM (MPa) | 6646 ± 481 | 6620 ± 419 | 6628 ± 436 | 6888 ± 893 | 7132 ± 590 | NE | NE |
| Shofu HC | Ra (µm) | 0.0446 ± 0.00996 | 0.0941 ± 0.00901 | 0.0232 ± 0.00303 | 0.1190 ± 0.01660 | 0.0290 ± 0.00310 | 0.0335 ± 0.01080 | 0.0330 ± 0.01087 |
|  | VH | 89.3 ± 1.69 | 85.3 ± 1.74 | 89.1 ± 1.99 | 78.7 ± 2.53 | 82.3 ± 1.32 | 89.5 ± 1.15 | 89.0 ± 0.995 |
|  | FS (MPa) | 181.3 ± 26.3 | 184.4 ± 13.0 | 184.5 ± 19.2 | 178.0 ± 19.7 | 193.7 ± 7.04 | NE | NE |
|  | EM (MPa) | 8598 ± 989 | 7768 ± 774 | 8697 ± 1711 | 8967 ± 1574 | 7997 ± 1004 | NE | NE |

RPL =periodic repolished group; DPC =double-polished control group; Ra =surface roughness; VH =Vickers microhardness; FS =flexural strength; EM =elastic modulus; NE =not evaluated.

**Note:** DPC groups were included only for surface roughness and Vickers microhardness analyses.
